# Closing the loop: 3C versus DNA FISH

**DOI:** 10.1186/s13059-016-1081-2

**Published:** 2016-10-19

**Authors:** Luca Giorgetti, Edith Heard

**Affiliations:** 1 Friedrich Miescher Institute for Biomedical Research, 4058 Basel, Switzerland; 2 Institut Curie, CNRS UMR3215, INSERM U934, Paris, Cedex 05 France; 3 Collège de France, Paris, 75005 France

## Abstract

Chromosome conformation capture (3C)-based techniques have revolutionized the field of nuclear organization, partly replacing DNA FISH as the method of choice for studying three-dimensional chromosome architecture. Although DNA FISH is commonly used for confirming 3C-based findings, the two techniques are conceptually and technically different and comparing their results is not trivial. Here, we discuss both 3C-based techniques and DNA FISH approaches to highlight their similarities and differences. We then describe the technical biases that affect each approach, and review the available reports that address their compatibility. Finally, we propose an experimental scheme for comparison of 3C and DNA FISH results.

## Introduction

Understanding the three-dimensional (3D) structure of chromosomes is a central focus in modern molecular biology. Besides characterizing how chromosome folding is achieved in order to package meters of DNA inside a cell nucleus that is only a few micrometers in diameter, it is important to understand how regulatory sequences interact with each other in 3D nuclear space [1, 2]. Moreover, in the context of a complex repetitive genome such as our own, in which only a fraction of DNA sequences actually participate directly in gene regulation, the challenge is to understand how specific regulatory elements can find and control relevant genes over long distances (often tens to thousands of kilobases away), somehow enabling expression in the right place and at the right time [3]. Furthermore, transcription can be a noisy process, generating variability that is probably essential in some contexts (such as dynamic developmental decisions) but that must be prevented in others [4]. How can these different scenarios be achieved in the context of chromatin dynamics—both in terms of the physical properties of chromatin as well as in biological processes such as the cell cycle, DNA replication, and so on? In addition, chromatin can be packaged very differently in the nucleus, with heterochromatin existing in many different states and occupying distinct and dynamic compartments, such as at the nuclear or nucleolar peripheries or in PML bodies [5]. Understanding how the genome is packaged and how this packaging is exploited or dealt with in different contexts presents important challenges.

Two experimental approaches that have been extensively used to investigate chromosome structure in eukaryotes are DNA fluorescent in situ hybridization (DNA FISH) [6] and chromosome conformation capture (3C) and its derivatives (reviewed in [7]). In the past, DNA FISH was the method of choice for investigations of the 3D structure of the genome. Despite being an intrinsically low-throughput technique that allows simultaneous evaluation of only a handful of genomic loci in parallel, DNA FISH has nevertheless allowed many fundamental discoveries to be made, such as the existence of chromosomal territories [8] and the dynamic repositioning of genomic loci with respect to nuclear compartments (such as the nuclear periphery) during differentiation (see [9] for a comprehensive review).

The recent advent of 3C-based approaches (such as circularized chromosome conformation capture (4C), chromosome conformation capture carbon copy (5C), and Hi-C [7]) has revolutionized the field of nuclear organization, enabling the detection of physical proximity between multiple genomic loci (and eventually across an entire genome) simultaneously. In this paper, we collectively refer to 3C-based techniques as “3C” for simplicity, since much of what we discuss is largely independent of which particular 3C-based variant is chosen. The development and refinement of 3C has led to several important discoveries, such as the compartmentalization of chromosomes into a complex hierarchy of folding levels, ranging from loops between sequences in the kilobase range [10], to sub-megabase topologically associating domains (TADs) that tend not to vary between tissues [11–14], and right up to multi-megabase active and inactive compartments [15], which vary between cell and tissue types. Thus, 3C technologies have transformed our view of the genome, and DNA FISH, which was once the state-of-the-art technique to study chromosome conformation, is increasingly regarded as an accessory tool that is used to confirm or validate 3C-based predictions.

In fact, DNA FISH and 3C are very different techniques that provide intrinsically different (and complementary) types of information. 3C-based approaches detect the cell population-averaged crosslinking probabilities of the chromatin fiber. The most recent versions of these techniques enable genome-wide, high-resolution measurements of the spatial proximity between genomic elements to be obtained. DNA FISH, on the other hand, enables the measurement of 3D distances between a limited number of genomic loci; it also enables the distribution of these distances within a cell population to be determined. This information is not accessible in 3C-based experiments. Moreover, the two techniques are affected by common, as well as specific, potential sources of experimental error. Nevertheless, with appropriate precautions and carefully designed experiments, the two methods can be powerfully combined to bring comprehensive insights into the folding of the genome over a wide range of length scales.

Several reviews have already covered recent studies that have explored the different scales of chromosome folding [1, 9, 16]. Here, we discuss the 3C and DNA FISH approaches commonly used in such studies from a rather theoretical perspective, with the aim of highlighting their similarities and differences and of suggesting a conceptual scheme for their comparison. We also describe the technical biases that can affect both techniques, reviewing the available reports that support (or question) their compatibility. Finally, we propose an experimental scheme for ensuring that the results of the two methods can be successfully compared in a coherent framework.

## 3D distances versus 3C counts: the theoretical link

DNA FISH usually involves fixation and permeabilization of cells, followed by hybridization of fluorescently labeled DNA probes (either single-stranded oligos or denatured double-stranded DNA) to specific loci on the chromatin fiber. Prior to hybridization, the sample must be slightly denatured to allow base-pairing to occur between the probe and the double-stranded target DNA. The target locus can then be directly visualized using fluorescence microscopy, enabling its localization to be assessed in the context of the overall nuclear architecture and/or with respect to other genomic loci [17]. Depending on the purpose of the experiment, different strategies for fixation, permeabilization, and imaging can be adopted. In two-dimensional (2D) DNA FISH [18, 19], the cells are swollen in hypotonic buffer and fixed with methanol and acetic acid in order to flatten the nuclei and thus allow 2D microscopy to be performed (without adjusting the focus in the Z direction). In 3D DNA FISH [17, 20], cellular morphology is maintained by fixation with formaldehyde and permeabilization with detergents such as Triton X-100. Cells maintain their 3D shape and imaging is performed in 3D (i.e., by acquiring stacks of individual 2D images in different focal planes). 2D FISH allows overall tendencies to be estimated and is largely sufficient in many cases (for example, for comparing chromosomal positioning, or positions of loci within chromosomes, between different cell types or between wild-type and mutant samples). It should be mentioned that 3D FISH is technically more challenging than 2D FISH, both in terms of the experimental complexity of the protocol and the more sophisticated imaging that it requires. For integrated comparison with 3C data, however, 3D FISH measurements are critical because they enable accurate distances to be calculated.

**Fig. 1:**
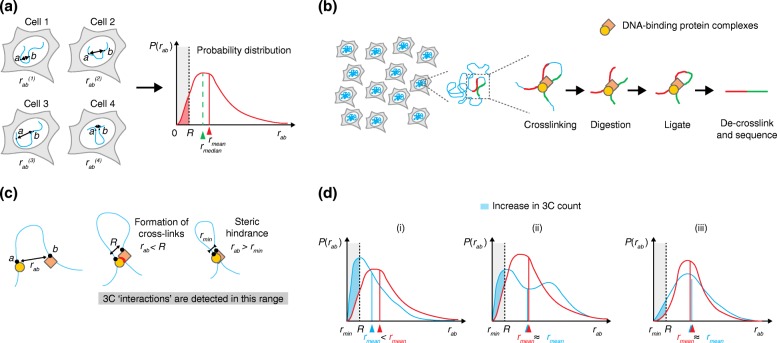
Comparison of 3C and DNA FISH experiments. **a** Cell-to-cell variation in the three-dimensional (3D) distance *r*_*ab*_ between two genomic loci *a* and *b* gives rise to the distribution of distances *P*(*r*_*ab*_). Knowledge of *P*(*r*_*ab*_) allows calculation of the mean and median 3D distances between *a* and *b r*_*mean*_ and *r*_*median*_. It also allows calculation of the fraction of cells for which the distance *r*_*ab*_ is smaller than a certain threshold *R.* **b** General scheme for 3C-based techniques. Cells are fixed and crosslinked chromatin is digested with a restriction endonuclease. Restriction fragments that were sufficiently close in the 3D space to be crosslinked by protein bridges (DNA-binding protein complexes) are then re-ligated to promote the formation of hybrid DNA molecules arising from 3D proximity events. De-crosslinked DNA is then sequenced to identify the ligated restriction fragments. The 3C signal for any locus pair (*a* and *b*) is proportional to the number of ligated restriction fragments that map to the two loci*.* **c** From a theoretical perspective, the 3C signal is generated by genomic loci whose 3D distance *r*_*ab*_ is smaller than the (locus-specific) crosslinking range (*R*) over which the two genomic loci can be crosslinked by formaldehyde, and bigger than a minimum distance *r*_*min*_ that arises from steric hindrance between the two parts of the fiber. **d** Hypothetical scenarios giving rise to an increase in 3C signal (*blue shaded area*) when comparing two distributions of 3D distances (*blue* versus *red curves*). (i) Increasing 3C signal results from an overall shift of distances towards smaller values, corresponding to decreased mean (or median) distance. (ii) 3C signal increases as a consequence of a shift from a mono- to a bimodal distribution of distances, for example, in the case of the appearance of two sub-populations in different conformational states (e.g., compact versus elongated). In this case *r*_*mean*_ (and *r*_*median*_, not shown) do not change. (iii) Increased 3C signal is a consequence of increased cell-to-cell variability in 3D distances, without appreciable changes in *r*_*mean*_ (or *r*_*median*_, not shown)

An advantage of 3D DNA FISH is that it allows a quantitative measurement of the *three-dimensional distance* (*r*_*ab*_) between two targeted loci *a* and *b* inside single cells. Consequently, the associated probability distribution *P*(*r*_*ab*_) that measures the variation of *r*_*ab*_ across the cell population can be extracted (Fig. 1a). Thus, DNA FISH allows us to estimate the distribution and degree of variability of 3D distances between pairs of genomic loci. In addition, useful statistics can be extracted from the probability distribution, such as the average or median 3D distance between two loci, or the number of cells with the distance between two loci below a certain threshold distance (Fig. 1a). Although mean or median distances are often used to describe DNA FISH measurements synthetically, quantitative measurements of cell-to-cell variation in 3D distances can provide valuable information on the underlying configurations of the chromatin fiber and have been used to infer possible models of chromosome folding [21].

On the other hand, 3C and its derivatives such as 4C, 5C, and Hi-C (reviewed in [7]), as well as Capture-C [22] and Capture-Hi-C [23], are population-averaged biochemical assays in which chromatin is digested with a restriction enzyme in fixed nuclei. Proximity-mediated ligation is then used to create hybrid DNA molecules between crosslinked restriction fragments whenever they are sufficiently close in the 3D space. It is important to stress that providing a clear definition of what is “sufficiently close” for crosslinking is not an easy task. As discussed in more detail below, it is reasonable to assume that chemical crosslinking through formaldehyde occurs between parts of the chromatin fiber that are separated by a range of 3D distances, from a few nanometers up to a few hundred nanometers. Despite the considerable uncertainty, these distances are typically smaller than average 3D distances between genomic loci considered in experiments comparing FISH and 3C-based techniques (often >1 μm). Thus, 3C and its derivatives can be thought of as detecting proximity events between genomic loci rather than their actual 3D distances.

Early implementations of 3C-based techniques relied on the detection of ligation products by PCR or microarrays, but the most recent versions employ high-throughput sequencing to detect proximity events and their abundance across the cell population [24]. The number of normalized read counts 3*C*_*ab*_ between loci *a* and *b* in Hi-C and 5C experiments can, in principle, be thought of as being proportional to the number of cells in which the two loci were located closer than a certain range *R* within which crosslinking occurs (which is most probably dominated by DNA-binding protein complexes; Fig. 1b). Thus, in principle, 3C-based read counts are linked to the distance distribution *P*(*r*_*ab*_) by the following relationship [25]:

1 $$ 3{C}_{ab}\approx \alpha \times {\displaystyle {\int}_{r_{min}}^R}4\pi\ {r}_{ab}^2P\left({r}_{ab}\right)\ d{r}_{ab} $$

where *α* is a proportionality constant, *r*_*min*_ is the minimum distance between loci *a* and *b* that can be reached due to steric repulsion between the two parts of the fiber, and *R* is the crosslinking range (Fig. 1c).

From this theoretical point of view (in which we disregard all sources of potential experimental biases), it is already clear that relating 3C-based counts with 3D distances measured by FISH is not a trivial task. Notably, the 3C signal is dominated by the low-distance portion of the distance distributions (between *r*_*min*_ and *R*). In support of this, 3C- and DNA FISH appear to be highly correlated when comparing 3C-based counts with colocalization of corresponding FISH probes, but only when defining colocalization radii that are much smaller (five- to tenfold) than the maximal distances measured by FISH [11, 26]. Therefore, changes in 3C count are not necessarily correlated to changes in average (mean) or median distances. The simplest case is when the 3C count increases as a consequence of decreased average distance between loci *a* and *b* (Fig. 1d(i)). This is frequently assumed to be the rule when comparing the results of 3C–DNA FISH experiments, and it seems reasonable to imagine that it is often the case. However, it must be noted that, in principle, the 3C signal can increase without a corresponding detectable increase in the average (or median) 3D distance between two loci. This is the case if the probability distribution of distances between loci *a* and *b* changes from unimodal to bimodal behavior (Fig. 1d(ii)), for example, as a consequence of the presence of two populations of cells in very different conformational states; or if the cell-to-cell variability in *r*_*ab*_ increases (Fig. 1d(iii)), for example, as a consequence of increased chromatin flexibility. In both cases, an experimental test of 3C results based on the detection of mean or median distances would fail. Thus, interpreting changes in 3C-based counts as changes in mean (or median) distances should not be the guideline when comparing experimental results obtained using the two techniques. Rather, we would suggest that an experimental scheme should rely on the detection of proximity or overlap between DNA FISH signals and a plot of their distributions, rather than on their mean or median distances (for examples, see [11, 26–29]).

As we have seen, relating the 3C count to the full distribution of distances through Eq. 1 is a complicated “inverse problem” that requires using data of reduced dimensionality (the two-dimensional matrix of 3C counts 3*C*_*ab*_) to infer the full distributions of 3D distances *P*(*r*_*ab*_), for all loci *a* and *b* considered in the experiment. In order to do this quantitatively, it is possible to use computational strategies based on coarse-grained physical models of the chromatin fiber, which try to find the 3D fiber conformations that are compatible with the set of restraints provided by the 3C results. To convert 3C counts into distances, all these models assume some sort of functional relationship between 3C count and the 3D distance of loci. Some models assume that the 3C count is inversely proportional to the mean 3D distance and produce an average conformation of a chromosomal region (for examples, see [30–33]); others assume that only a fraction of conformations in a population for which the distance *r*_*ab*_ is smaller than a crosslinking range *R* contribute to the 3C signal, as in Eq. 1 (see [28, 34]). Although the former class of models can be useful when building a schematic, simplified conformational representation of the genomic region under study, the latter are likely to provide more realistic ensembles of conformations. These population-based models are a useful tool in comparing 3C and DNA FISH data because they allow the actual distribution of distances *P*(*r*_*ab*_) to be deconvolved out of the 3C data and to be compared with high-resolution DNA FISH experiments [28, 34].

## Experimental biases affecting 3C and DNA FISH

Both 3C and DNA FISH experiments are subject to a range of technical issues. Some of these impact the two techniques in a comparable manner, which facilitates comparison of the results obtained, whereas other technical issues are specific to one or the other or affect the two approaches differently. One fundamental similarity (and common bias) is that both DNA FISH and 3C are based on chemical crosslinking with formaldehyde. Formaldehyde crosslinking favors DNA–protein [35] and, to a much greater extent, protein–protein interactions with a bias towards lysine, tryptophan, and cysteine [36]. Although crosslinking occurs across a range of 2–3 Å [37], DNA loci that are much more distant are indirectly crosslinked through the creation of protein–protein networks [38]. Hence, irrespective of whether the interaction events detected by 3C arise because of direct (molecular) or indirect (protein-bridge mediated) proximity [39], these de facto distances at which the proximity events occur can also be measured in a DNA FISH experiment (notably if the latter is performed in the same crosslinking conditions and in non-perturbative denaturation conditions). Specifically, proximity events constitute the low-distance tail of the distribution of distances *P*(*r*_*ab*_) between two loci *a* and *b* (Eq. 1).

A second similarity is that both DNA FISH and 3C require that nuclei are permeabilized in order to allow fluorescent probes or restriction enzymes to access the chromatin. Permeabilization is usually achieved by treatment with detergents: Triton X-100 (in FISH) or Igepal CA-630 (in 3C). Although this step might be thought to lead to perturbations in 3D nuclear structure, both electron and high-resolution optical microscopies have shown that nuclear ultrastructure is well conserved, at least upon Triton permeabilization [40, 41]. Alternative approaches that better conserve nuclear ultrastructure by avoiding permeabilization have been developed. These include CryoFISH [42], in which probe hybridization occurs on cryosections of sucrose-embedded cells; however, the 2D sectioning used in this technique does not allow the determination of 3D distances unless consecutive sections from the same cells are analyzed.

Aside from these similarities, the 3C and DNA FISH techniques also have specific technical issues that need to be evaluated accurately and controlled in order to ensure that the results can be correctly interpreted. In 3D DNA FISH, crosslinked chromatin must be mildly denatured in order to allow base-pairing between the probe and the target DNA. This is achieved by heating the sample in the presence of formamide, which reduces the melting temperature of double-stranded DNA and allows DNA denaturation at lower temperatures than in aqueous solution. Heat denaturation is, of course, a major source of concern in DNA FISH experiments because it may lead to changes in nuclear and chromatin organization. Indeed, electron microscopy observations [43] have shown that heat denaturation can induce appreciable changes in the fine-scale structure of the chromatin network. It is unclear to what extent the 3D distances (>100 nm on average) between genomic loci that are usually detected in DNA FISH (>50 kb apart) are altered as a consequence of denaturation. Experiments detecting LacO-tagged chromatin loci by using a Lac repressor protein fused to green fluorescent protein (GFP) in undenatured cells have given somewhat reassuring results, with FISH signals in structurally preserved cells showing a surprising similarity to signals generated before denaturation, at least at the scale of hundreds of nanometers [44]. With the improving resolution of 3C-based experiments [10, 22], a careful evaluation of denaturation artifacts will be essential to ensure that the comparison of 3C and DNA FISH results is meaningful. The recently developed CASFISH technique [45], which relies on the binding of fluorescent Cas9 fusion proteins to crosslinked (but not denatured) chromatin in cell nuclei, could provide an interesting tool with which to compare 3D distances before or after denaturation, if combined with standard 3D DNA FISH experiments on the same loci performed sequentially in the same cells.

Another important bias that can influence DNA FISH measurements occurs at the level of fluorescence microscopy and imaging. A common but often overlooked issue concerns chromatic and mechanical aberrations that result in artifactual displacements between signals generated by probes that are labeled with different fluorophores. Although this effect is usually negligible for loci that are separated by large genomic distances, chromatic and mechanical aberrations can substantially affect the measurement of distances between loci that lie in the tens to hundreds of kilobases range (when physical distances approach a few hundred nanometers). Such aberrations have to be taken into account and can be corrected for computationally [46] to ensure that the distribution of distances *P*(*r*_*ab*_) in Eq. 1 is correctly sampled. This is especially important for the small distances that dominate the 3C signal. The amount of aberrations present in a microscopy setup can be easily estimated by using calibration beads labeled in multiple fluorophores and then measuring the shift in the positions of bead centers when beads are imaged in the various colors. Correction of such aberrations can be performed by elastic registration of DNA FISH images. Various methods that can be used to achieve this have been implemented in user-friendly computer programs such as ImageJ (for example, see [47, 48]).

The 3C-based techniques are also characterized by certain distinct technical issues that can affect the experimental procedure and impact on how the data are analyzed, especially when they are to be compared to DNA FISH data. Such biases include the efficiency of chromatin digestion, the choice of restriction enzyme used, the efficiency of ligation, and other biases (see [49, 50] for details). Here, we focus on two main issues that are directly relevant to comparisons of 3C data with DNA FISH measurements.

First, the range *R* over which two genomic loci can be crosslinked by formaldehyde (Fig. 1b, c and Eq. 1) is not known. Quantitative comparisons between 4C or 5C reads and percentage of colocalizing FISH signals give optimal agreement when colocalization is defined in a range of 500 nm–1 μm [11, 26]; calculations based on modeling and comparison with DNA FISH measurements have estimated the crosslinking range to be approximately 100 nm [28]. This value could depend, however, on the local concentration of amino groups that are responsible for formaldehyde-mediated crosslinking. It is thus possible (although formally unproven) that this value could be modulated by the local chromatin composition [51] and by indirect cross-linking to large insoluble nuclear aggregates [38].

## How should 3C and DNA FISH be compared?

Given these various technical issues and possible experimental biases, it seems legitimate to question whether the results of 3C-based techniques can be compared to (or even validated by) DNA FISH measurements.

Although negative (or discordant) 3C versus FISH correlations may be underrepresented in the literature because of an obvious publication bias towards positive reports, the majority of studies suggest that 3C-based techniques and 3D DNA FISH give concordant results over a wide range of genomic and spatial ranges [10, 11, 26–28, 34, 52–54]. Moreover, this seems to be the case irrespective of which variant of the 3C method was employed (4C, 5C, Hi-C, or Capture-C). This suggests that the technical issues that specifically affect either of the two techniques probably have a relatively small impact, except perhaps at some specific locations in the genome. For example, clearly discordant outcomes of 3C and DNA FISH have been reported at the HoxD cluster [51]. At this locus, the chromatin state differs massively between the two biological conditions investigated in the study, namely Polycomb repressive complex 1 (PRC1) and PRC2 knockout mouse embryonic stem cells. In PRC1 mutant (*Ring1B−/−*) cells, the HoxD locus remains associated with high levels of PRC2 proteins, whereas in PRC2 (*Eed−/−*) mutant cells, the PRC2 coating is lost. Williamson et al. [51] reported concordant 5C and FISH results in PRC2 (*Eed−/−*) cells, where both techniques pointed to chromatin unfolding, when compared to wild-type cells. However, in PRC1 (*Ring1B−/−*) cells, discordant results were observed; here, strong 5C interactions (unaltered compared to those of wild-type cells) were contradicted by decompaction observed in FISH. The authors suggested that the observed 5C versus FISH discrepancy could result from differential crosslinking efficiencies when large quantities of PRC2 are lost (*Eed−/−*) or maintained (*Ring1b−/−*) at the HoxD locus. Thus, it is possible that, in certain specific cases, where the chromatin composition changes in a major way between experimental samples, the FISH and 3C-based results may diverge. Another study on the same locus, the physical compaction at the HoxD locus was measured in vivo by DNA FISH and super-resolution microscopy [55]. However, the study examined different tissues of wild-type mice in which the changes in chromatin composition are milder than those in PRC1 versus PRC2 knock-out cells. In this case, the compaction measured by FISH was correctly predicted by 4C-based experiments. Thus, the divergence between DNA FISH and 3C techniques seems to occur mainly when there are relatively massive changes in chromatin content at a particular locus, and this is clearly something that should be borne in mind when comparative 3C versus DNA FISH experiments are performed.

An important aspect that needs to be taken into consideration when comparing DNA FISH and 3C results is the choice of the criteria that have to be used to quantify DNA FISH distance distributions. Since short-range distances below the crosslinking range *R* dominate the 3C count (Eq. 1), methods that detect physical proximity rather than mean 3D distances should be more suited for comparisons between 3C and DNA FISH results. In fact, colocalization methods that are based on correlations of pixel intensities [11, 27] have proven to be very sensitive in detecting subtle differences in contact probabilities, such as those resulting from changes that occur inside or outside of TADs, predicted by 3C approaches. This may result from the fact that these methods assess overlaps between relatively extended objects rather than absolute 3D distances, and thus may be more sensitive in detecting short-distance features such as partial overlaps between signal peripheries.

On the other hand, measuring 3D distances is clearly the method of choice for either assessing difference in structure over large genomic distances [26] or testing quantitatively the predictions of structural models [28, 34]. It is important to keep in mind, however, that the full probability distribution of distances (and not just the mean) must be carefully sampled in order to avoid misleading interpretations.

In conclusion, we propose an experimental scheme for the careful comparison of 3C and DNA FISH results to ensure that the validity of the comparison is maximized:

1. To minimize the effect of potential fixation biases, fixation conditions for 3C and FISH should be as similar as possible. Possibly, a fraction of the same cells that were fixed for 3C should be separated from the main batch and used immediately to prepare coverslips for DNA FISH (this can be done, for example, by adsorbing fixed cells on poly-L-lysine-coated coverslips). Alternatively, another batch of cells grown on coverslips should be fixed with the reagents used for 3C.
2. When comparing different conditions in DNA FISH (such as different differentiation stages or mutants), the various samples should be processed in parallel in order to minimize differences in denaturation time and temperature. Ideally, all samples should be dispensed on the same coverslip to ensure maximal uniformity of conditions.
3. Optical aberrations should be limited as much as possible during image acquisition. Mechanical stage drifts, which can result in image distortions, should be minimized (for example, by using stabilized sample stages). Chromatic aberrations should be reduced by preferring microscopes that mount filter wheels in combination with multiband-pass dichroic mirrors, instead of rotating filter cubes [46].
4. Despite all precautions and even in stable and well-aligned microscopes, residual aberrations can be of the order of a few hundreds of nanometers, and thus comparable to the distances that dominate the 3C signal. These residual aberrations need to be measured using fluorescent beads and computationally corrected after image acquisition using elastic channel registration [46] in order to make sure that short distances (below 300 nm) are correctly sampled.
5. Replicate 3C-based experiments should be performed in parallel to account for technical and biological variability correctly [56].
6. 3C counts should not be exclusively compared to mean 3D distances measured by DNA FISH. We suggest that different methods should be used in parallel to compare the two techniques, notably those that measure spatial proximity (for example, correlation of pixel intensities).

Although it is impossible to rule out confounding effects resulting from the specific bias of each technique completely, these expedients should at least ensure that the comparison between 3C-based results and DNA FISH measurements is fair.

## Conclusions

Accurately designed combined 3C–DNA FISH experiments represent a powerful tool that can be used to determine the 3D conformation of chromosomes, including its cell-to-cell variability and its relevance in the context of fundamental biological functions such as transcription. Further, the possibility of combining DNA FISH with super-resolution microscopy [11, 55, 57] and/or integrating it with single-cell 3C approaches [58] opens exciting new avenues. However, one major drawback of these crosslinking-based techniques is that they do not allow assessment of the temporal dynamics that underlie the cell-to-cell variability in chromosome conformation. An ultimate level of understanding of chromosomal structures, and notably an understanding of their cell-to-cell and temporal variability, will thus undoubtedly come from live-cell experiments in which (groups of) single chromosome loci can be imaged simultaneously and over many cells. This has proven to be a daunting task, especially in mammalian cells, and has been achieved in the past by using homologous recombination to insert exogenous genomic sequences that can be visualized by fluorescent bacterial operators [59]. The genome engineering revolution [60] has brought this perspective closer by enabling the easier generation of knock-in mammalian cell lines [61]. Engineered cell lines could provide recruitment sites for fluorescent operators or oligomerizing proteins [62], or could even allow recruitment of multiple fluorophores to endogenous DNA directly using catalytically inactive Cas9 [63].

Live-cell imaging is likely to remain a low-throughput technique that allows the investigation of a small number of genomic loci in tightly controlled experimental systems, such as clonal culture cell lines expressing controlled levels of fluorescent proteins. In developmental or disease-related contexts (for example, in human tissues), 3C-based approaches complemented with DNA FISH will probably remain the methods of choice for the investigation of chromatin structure. It will therefore be crucial to implement accurately controlled 3C–FISH experiments to compare the results of these two techniques, notably by adopting the procedures we have proposed in this review. In addition, novel approaches to 3C and FISH that do not require crosslinking, such as native 3C [64] and CASFISH [45], may pave the way to more physiological experiments that combine population-averaged and single-cell techniques.

Finally, thanks to the greatly improved ability to modify mammalian genomes with CRISPR/Cas9 or TALEN approaches, we are entering an era when the structural and functional models of chromatin organization that can be built on the basis of 3C and FISH results can be tested genetically. On the one hand, this will require carefully controlled quantitative experiments, but on the other hand, it will provide an unprecedented opportunity to obtain a mechanistic description of chromosome conformation and its function in the context of transcription and other important biological processes.

## Abbreviations

3C: Chromosome conformation capture
3D: Three-dimensional
4C: Circularized chromosome conformation capture
5C: Chromosome conformation capture carbon copy
FISH: Fluorescence in situ hybridization
PRC1: Polycomb repressive complex1
TAD: topologically associating domain

## References

[1] Bouwman BA, de Laat W (2015). Getting the genome in shape: the formation of loops, domains and compartments. Genome Biol.

[2] Dekker J, Mirny L (2016). The 3D genome as moderator of chromosomal communication. Cell.

[3] Spitz F (2016). Gene regulation at a distance: from remote enhancers to 3D regulatory ensembles. Semin Cell Dev Biol.

[4] Balázsi G, van Oudenaarden A, Collins JJ (2011). Cellular decision making and biological noise: from microbes to mammals. Cell.

[5] Belmont A (2003). Dynamics of chromatin, proteins, and bodies within the cell nucleus. Curr Opin Cell Biol.

[6] Bridger JM, Volpi EV (2010). Fluorescence in situ hybridization (FISH): protocols and applications.

[7] de Wit E, de Laat W (2012). A decade of 3C technologies: insights into nuclear organization. Genes Dev.

[8] Cremer T, Cremer C (2001). Chromosome territories, nuclear architecture and gene regulation in mammalian cells. Nat Rev Genet.

[9] Fraser J, Williamson I, Bickmore WA, Dostie J (2015). An overview of genome organization and how we got there: from FISH to Hi-C. Microbiol Mol Biol Rev.

[10] Rao SSP, Huntley MH, Durand NC, Stamenova EK, Bochkov ID, Robinson JT (2015). A 3D map of the human genome at kilobase resolution reveals principles of chromatin looping. Cell.

[11] Nora EP, Lajoie BR, Schulz EG, Giorgetti L, Okamoto I, Servant N (2012). Spatial partitioning of the regulatory landscape of the X-inactivation centre. Nature.

[12] Dixon JR, Selvaraj S, Yue F, Kim A, Li Y, Shen Y (2012). Topological domains in mammalian genomes identified by analysis of chromatin interactions. Nature.

[13] Hou C, Li L, Qin ZS, Corces VG (2012). Gene density, transcription, and insulators contribute to the partition of the *Drosophila* genome into physical domains. Mol Cell.

[14] Sexton T, Yaffe E, Kenigsberg E, Bantignies F, Leblanc B, Hoichman M (2012). Three-dimensional folding and functional organization principles of the *Drosophila* genome. Cell.

[15] Lieberman-Aiden E, van Berkum NL, Williams L, Imakaev M, Ragoczy T, Telling A (2009). Comprehensive mapping of long-range interactions reveals folding principles of the human genome. Science.

[16] Gibcus JH, Dekker J (2013). The hierarchy of the 3D genome. Mol Cell.

[17] Chaumeil J, Augui S, Chow JC, Heard E, Hancock R (2008). Combined immunofluorescence, RNA fluorescent in situ hybridization, and DNA fluorescent in situ hybridization to study chromatin changes, transcriptional activity, nuclear organization, and X-chromosome inactivation. The nucleus.

[18] Bickmore WA, Carothers AD (1995). Factors affecting the timing and imprinting of replication on a mammalian chromosome. J Cell Sci.

[19] Garimberti E, Tosi S, Bridger JM, Volpi EV (2010). Fluorescence in situ hybridization (FISH), basic principles and methodology. Fluorescence in situ hybridization (FISH).

[20] Cremer M, Müller S, Köhler D, Brero A, Solovei I (2007). Cell preparation and multicolor FISH in 3D preserved cultured mammalian cells. Cold Spring Harb Protoc.

[21] Sachs RK, van den Engh G, Trask B, Yokota H, Hearst JE (1995). A random-walk/giant-loop model for interphase chromosomes. Proc Natl Acad Sci U S A.

[22] Davies JOJ, Telenius JM, McGowan SJ, Roberts NA, Taylor S, Higgs DR (2016). Multiplexed analysis of chromosome conformation at vastly improved sensitivity. Nat Methods.

[23] Martin P, McGovern A, Orozco G, Duffus K, Yarwood A, Schoenfelder S (2015). Capture Hi-C reveals novel candidate genes and complex long-range interactions with related autoimmune risk loci. Nat Commun.

[24] Belton J-M, McCord RP, Gibcus JH, Naumova N, Zhan Y, Dekker J (2012). Hi–C: a comprehensive technique to capture the conformation of genomes. Methods.

[25] Rosa A, Becker NB, Everaers R (2010). Looping probabilities in model interphase chromosomes. Biophys J.

[26] Hakim O, Sung M-H, Voss TC, Splinter E, John S, Sabo PJ (2011). Diverse gene reprogramming events occur in the same spatial clusters of distal regulatory elements. Genome Res.

[27] Crane E, Bian Q, McCord RP, Lajoie BR, Wheeler BS, Ralston EJ (2015). Condensin-driven remodelling of X chromosome topology during dosage compensation. Nature.

[28] Giorgetti L, Galupa R, Nora EP, Piolot T, Lam F, Dekker J (2014). Predictive polymer modeling reveals coupled fluctuations in chromosome conformation and transcription. Cell.

[29] Giorgetti L, Lajoie BR, Carter AC, Attia M, Zhan Y, Xu J (2016). Structural organization of the inactive X chromosome in the mouse. Nature.

[30] Duan Z, Andronescu M, Schutz K, McIlwain S, Kim YJ, Lee C (2010). A three-dimensional model of the yeast genome. Nature.

[31] Baù D, Sanyal A, Lajoie BR, Capriotti E, Byron M, Lawrence JB (2011). The three-dimensional folding of the α-globin gene domain reveals formation of chromatin globules. Nat Struct Mol Biol.

[32] Marbouty M, Le Gall A, Cattoni DI, Cournac A, Koh A, Fiche J-B (2015). Condensin- and replication-mediated bacterial chromosome folding and origin condensation revealed by Hi-C and super-resolution imaging. Mol Cell.

[33] Lesne A, Riposo J, Roger P, Cournac A, Mozziconacci J (2014). 3D genome reconstruction from chromosomal contacts. Nat Methods.

[34] Brackley CA, Brown JM, Waithe D, Babbs C, Davies J, Hughes JR (2016). Predicting the three-dimensional folding of cis-regulatory regions in mammalian genomes using bioinformatic data and polymer models. Genome Biol.

[35] Lu K, Ye W, Zhou L, Collins LB, Chen X, Gold A (2010). Structural characterization of formaldehyde-induced cross-links between amino acids and deoxynucleosides and their oligomers. J Am Chem Soc.

[36] Toews J, Rogalski JC, Clark TJ, Kast J (2008). Mass spectrometric identification of formaldehyde-induced peptide modifications under in vivo protein cross-linking conditions. Anal Chim Acta.

[37] Sutherland BW, Toews J, Kast J (2008). Utility of formaldehyde cross-linking and mass spectrometry in the study of protein–protein interactions. J Mass Spectrom.

[38] Gavrilov A, Razin SV, Cavalli G (2015). In vivo formaldehyde cross-linking: it is time for black box analysis. Brief Funct Genomics.

[39] Belmont AS (2014). Large-scale chromatin organization: the good, the surprising, and the still perplexing. Curr Opin Cell Biol.

[40] Solovei I, Cremer M, Bridger JM, Volpi EV (2010). 3D-FISH on cultured cells combined with immunostaining. Fluorescence in situ hybridization (FISH).

[41] Markaki Y, Smeets D, Cremer M, Schermelleh L. Fluorescence in situ hybridization applications for super-resolution 3D structured illumination microscopy. In: Sousa AA, Kruhlak MJ, editors. Nanoimaging. New York City: Humana Press; 2013. p. 43–64. http://dx.doi.org/10.1007/978-1-62703-137-0_4. 10.1007/978-1-62703-137-0_4 23086869

[42] Xie SQ, Lavitas L-M, Pombo A, Bridger JM, Volpi EV (2010). CryoFISH: fluorescence in situ hybridization on ultrathin cryosections. Fluorescence in situ hybridization (FISH).

[43] Solovei I, Cavallo A, Schermelleh L, Jaunin F, Scasselati C, Cmarko D (2002). Spatial preservation of nuclear chromatin architecture during three-dimensional fluorescence in situ hybridization (3D-FISH). Exp Cell Res.

[44] Kim I-H, Nagel J, Otten S, Knerr B, Eils R, Rohr K (2007). Quantitative comparison of DNA detection by GFP-lac repressor tagging, fluorescence in situ hybridization and immunostaining. BMC Biotechnol.

[45] Deng W, Shi X, Tjian R, Lionnet T, Singer RH (2015). CASFISH: CRISPR/Cas9-mediated in situ labeling of genomic loci in fixed cells. Proc Natl Acad Sci U S A.

[46] Giorgetti L, Piolot T, Heard E, Nakagawa S, Hirose T (2015). High-resolution 3D DNA FISH using plasmid probes and computational correction of optical aberrations to study chromatin structure at the sub-megabase scale. Nuclear bodies and noncoding RNAs.

[47] Sorzano COS, Thevenaz P, Unser M (2005). Elastic registration of biological images using vector-spline regularization. IEEE Trans Biomed Eng.

[48] Wang C-W, Ka S-M, Chen A (2014). Robust image registration of biological microscopic images. Sci Rep.

[49] Yaffe E, Tanay A (2011). Probabilistic modeling of Hi-C contact maps eliminates systematic biases to characterize global chromosomal architecture. Nat Genet.

[50] Nagano T, Várnai C, Schoenfelder S, Javierre B-M, Wingett SW, Fraser P (2015). Comparison of Hi-C results using in-solution versus in-nucleus ligation. Genome Biol.

[51] Williamson I, Berlivet S, Eskeland R, Boyle S, Illingworth RS, Paquette D (2014). Spatial genome organization: contrasting views from chromosome conformation capture and fluorescence in situ hybridization. Genes Dev.

[52] Splinter E, de Wit E, Nora EP, Klous P, van de Werken HJG, Zhu Y (2011). The inactive X chromosome adopts a unique three-dimensional conformation that is dependent on Xist RNA. Genes Dev.

[53] Chaumeil J, Micsinai M, Ntziachristos P, Roth DB, Aifantis I, Kluger Y (2013). The RAG2 C-terminus and ATM protect genome integrity by controlling antigen receptor gene cleavage. Nat Commun.

[54] Kalhor R, Tjong H, Jayathilaka N, Alber F, Chen L (2012). Genome architectures revealed by tethered chromosome conformation capture and population-based modeling. Nat Biotechnol.

[55] Fabre PJ, Benke A, Joye E, Huynh THN, Manley S, Duboule D (2015). Nanoscale spatial organization of the HoxD gene cluster in distinct transcriptional states. Proc Natl Acad Sci U S A.

[56] Naumova N, Imakaev M, Fudenberg G, Zhan Y, Lajoie BR, Mirny LA (2013). Organization of the mitotic chromosome. Science.

[57] Boettiger AN, Bintu B, Moffitt JR, Wang S, Beliveau BJ, Fudenberg G (2016). Super-resolution imaging reveals distinct chromatin folding for different epigenetic states. Nature.

[58] Nagano T, Lubling Y, Stevens TJ, Schoenfelder S, Yaffe E, Dean W (2013). Single-cell Hi-C reveals cell-to-cell variability in chromosome structure. Nature.

[59] Masui O, Bonnet I, Le Baccon P, Brito I, Pollex T, Murphy N (2011). Live-cell chromosome dynamics and outcome of X chromosome pairing events during ES cell differentiation. Cell.

[60] Sternberg SH, Doudna JA (2015). Expanding the biologist’s toolkit with CRISPR-Cas9. Mol Cell.

[61] Flemr M, Bühler M (2015). Single-step generation of conditional knockout mouse embryonic stem cells. Cell Rep.

[62] Saad H, Gallardo F, Dalvai M, Tanguy-le-Gac N, Lane D, Bystricky K (2014). DNA dynamics during early double-strand break processing revealed by non-intrusive imaging of living cells. PLoS Genet.

[63] Chen B, Gilbert LA, Cimini BA, Schnitzbauer J, Zhang W, Li G-W (2013). Dynamic imaging of genomic loci in living human cells by an optimized CRISPR/Cas system. Cell.

[64] Melnik S, Deng B, Papantonis A, Baboo S, Carr IM, Cook PR (2011). The proteomes of transcription factories containing RNA polymerases I,II or III. Nat Methods.

